# Metabolomics reveals variation and correlation among different tissues of olive (*Olea europaea* L.)

**DOI:** 10.1242/bio.025585

**Published:** 2017-07-31

**Authors:** Rao Guodong, Liu Xiaoxia, Zha Weiwei, Wu Wenjun, Zhang Jianguo

**Affiliations:** 1 State Key Laboratory of Tree Genetics and Breeding, Research Institute of Forestry, Chinese Academy of Forestry, Beijing 100091, China; 2 Collaborative Innovation Center of Sustainable Forestry in Southern China, Nanjing Forestry University, Nanjing 210037, China; 3 Key Laboratory of Tree Breeding and Cultivation, State Forestry Administration, Research Institute of Forestry, Chinese Academy of Forestry, Beijing 100091, China; 4 Gansu Academy of Forestry, Lanzhou 730030, China

**Keywords:** Metabolome, Olive, Different tissue, ANOVA, Metabolite-metabolite, Correlation

## Abstract

Metabolites in olives are associated with nutritional value and physiological properties. However, comprehensive information regarding the olive metabolome is limited. In this study, we identified 226 metabolites from three different tissues of olive using a non-targeted metabolomic profiling approach, of which 76 named metabolites were confirmed. Further statistical analysis revealed that these 76 metabolites covered different types of primary metabolism and some of the secondary metabolism pathways. One-way analysis of variance (ANOVA) statistical assay was performed to calculate the variations within the detected metabolites, and levels of 65 metabolites were differentially expressed in different samples. Hierarchical cluster analysis (HCA) dendrograms showed variations among different tissues that were similar to the metabolite profiles observed in new leaves and fruit. Additionally, 5776 metabolite-metabolite correlations were detected by a Pearson correlation coefficient approach. Screening of the calculated correlations revealed 3136, 3025, and 5184 were determined to metabolites and had significant correlations in three different combinations, respectively. This work provides the first comprehensive metabolomic of olive, which will provide new insights into understanding the olive metabolism, and potentially help advance studies in olive metabolic engineering.

## Abstract (precis)

**Summary:** This work provides the first comprehensive metabolomic of olive, which will provide new insights into understanding the olive metabolism and potentially help advance studies in olive metabolic engineering.

## INTRODUCTION

The olive (*Olea europaea* L.) belongs to the family Oleaceae, which is native to tropical and warm temperate regions, such as the Mediterranean. Archaeological and molecular data showed that the first cultivars originated 6000 years ago in Levant, a region currently located at the border between southwestern Turkey and northwestern Syria (Kaniewski et al., 2012; Ali et al., 2014). Olive trees are widely distributed throughout the world and cultivated commercially throughout Australia, South Africa, North and South America, and China; however, 98% of all olive trees are located in the Mediterranean Basin. Olive trees were introduced in China in the 1960s, and there are now over 200 cultivars planted in three major provinces (Gansu, Sichuan, and Yunnan) of China.

The olive tree is essential for the production of olive oil, which contain many kinds of unsaturated fatty acids and polyphenol (Özcan and Matthäus, 2016). Polyunsaturated fatty acids (PUFAs), which are regarded as an indispensable component of cell structure and development, are essential fatty acids that cannot be synthesized by the human body. The main PUFAs in olive oil are oleic acid (C_18_:1), palmitoleic acid (C_16_:1), linoleic acid (C_18_:2), and linolenic acid (C_18_:3). Olive oil is mainly concentrated in the pericarp (96%–98%), which results in its having a unique flavor and fragrance; accordingly, it is widely used for food preparations (Gavriilidou and Boskou, 1991). In Mediterranean countries, olive oil is the main dietary fat and is considered to be one the healthiest foods because of its strong association with reduced incidence of cardiovascular diseases and certain cancers (Trichopoulou et al., 2003). Byproducts that are extracted from olive oil and olive leaves also have a long history of medicinal value (Soler-Rivas et al., 2000). The most important byproducts of olives are plant phenolic compounds, which are well known to be involved in the response to stress conditions such as UV radiation, wounding, and infection (Tuck and Hayball, 2002). Olive oil phenolics are antioxidant compounds that have been shown to have antioxidant activities, and play a role in the delay of progression of atherosclerosis in animal systems (Marrugat et al., 2004). Moreover, several investigations have confirmed that olive oil helps decrease blood pressure (Mensink et al., 1988; Rasmussen et al., 1993; Fitó et al., 2005).

The compounds involved in olive metabolism have been investigated in several studies. A total of 475 Sicilian virgin olive oils produced during 10 different crop years (from 1993 to 2004) were studied for their fatty acids composition using the official gas chromatographic method, which demonstrated it is possible to employ an official and inexpensive analytical method coupled with the statistical analysis to ascertain the geographical origin and cultivar of an extra virgin olive oil (Di Bella et al., 2007). Analysis of the physicochemical properties, stability and the fatty acid, triacylglycerol, sterol, and triterpenic dialcohol compositions of six Tunisian olive oil varieties were analyzed and revealed significant differences between oil samples and great variability in the oil composition between cultivars (Haddada et al., 2007). Analysis of the triglycerides, total and two-position fatty acid composition of the economically important Cornicabra virgin olive oil variety from several consecutive crop seasons revealed that they were suitable for satisfactory classification of virgin olive oil extracted from Spanish olive varieties (Aranda et al., 2004). However, no comprehensive studies of the dynamic metabolite changes in different tissues of olive trees have been published to date. Metabolomic analysis involves detecting and quantifying metabolic changes with techniques such as nuclear magnetic resonance (NMR) spectroscopy and mass spectrometry, and integrating the resulting data with multivariate statistical techniques such as principal component analysis (PCA) and orthogonal signal correction projection to latent structure discriminant analysis (OPLS-DA) (Zhang et al., 2011). In this study, a gas chromatography-mass spectrometry (GC-MS)-based metabolomic approach was utilized to investigate the metabolic composition and natural metabolite variations in different tissues of olive tree (olive cultivar: Leccino). Cluster analysis, PCA and one-way analysis of variance (ANOVA) analyses of metabolites in olive leaves and fruit were studied, and metabolite-metabolite correlation analysis was performed. The results of this study will provide new insights into the understanding of metabolite shifts among different tissues of olive trees.

## RESULTS AND DISCUSSION

### Metabolomic profiling of olive leaves and fruit

**Fig. 1.**
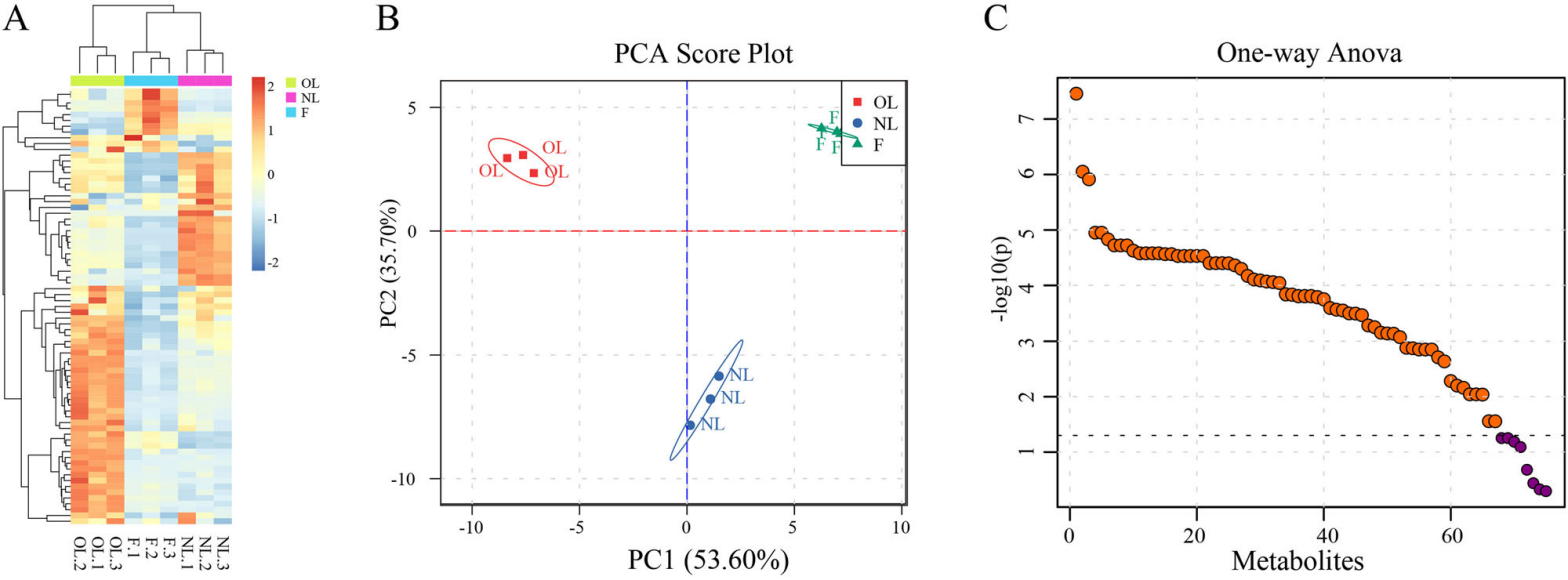
**Cluster analysis, PCA and one-way ANOVA of metabolites in olive leaves and fruit.** (A) Heat map representation of 76 metabolites. (B) PCA scores plot generated from all 76 metabolites of different samples. (C) Metabolites identified as statistically significant (*P*<0.05, dotted line; Tukey's multiple comparison test; data represented as mean±s.d.) are shown in orange, while non-significant metabolites are shown in purple.

An untargeted global metabolomics platform with GC-MS was used for olive metabolic profiling. A total of 226 metabolites were detected, of which 76 named metabolites were confirmed using the National Institute of Standards and Technology (NIST) and Wiley libraries. These metabolites covered different primary metabolism pathways. We then performed hierarchical clustering analysis to classify the 76 identified metabolites. Seven major metabolite groups were classified (Fig. 1A), with the largest containing 31 organic acid metabolites and 40.79% of the total number of identified metabolites. The second largest group had 17 metabolites involved in carbohydrate metabolism and 22.37% of the total identified metabolites. The third largest group (15.79%) consisted of 12 metabolites related to polyol metabolism, followed by five (6.58%) metabolites involved in phosphates metabolism, four (5.26%) in fatty acids metabolism, and one (1.32%) in amino acids metabolism. PCA of all 76 metabolites was conducted to compare their metabolic compositions, and two principal components were found to explain 89.30% of the overall variance of metabolite profiles (53.60% and 35.70% for PC1 and PC2, respectively; Fig. 1B). A one-way ANOVA statistical assay was performed to calculate the variations within the detected metabolites, and levels of 65 metabolites were differentially expressed at the different samples (Fig. 1C). These 65 metabolites were from leaves and fruits and included 27 organic acids, 17 carbohydrates, 11 polyols, four phosphoric acids, two fatty acids and one amino acid.

### Metabolic variations in olive leaves and fruit

**Fig. 2.**
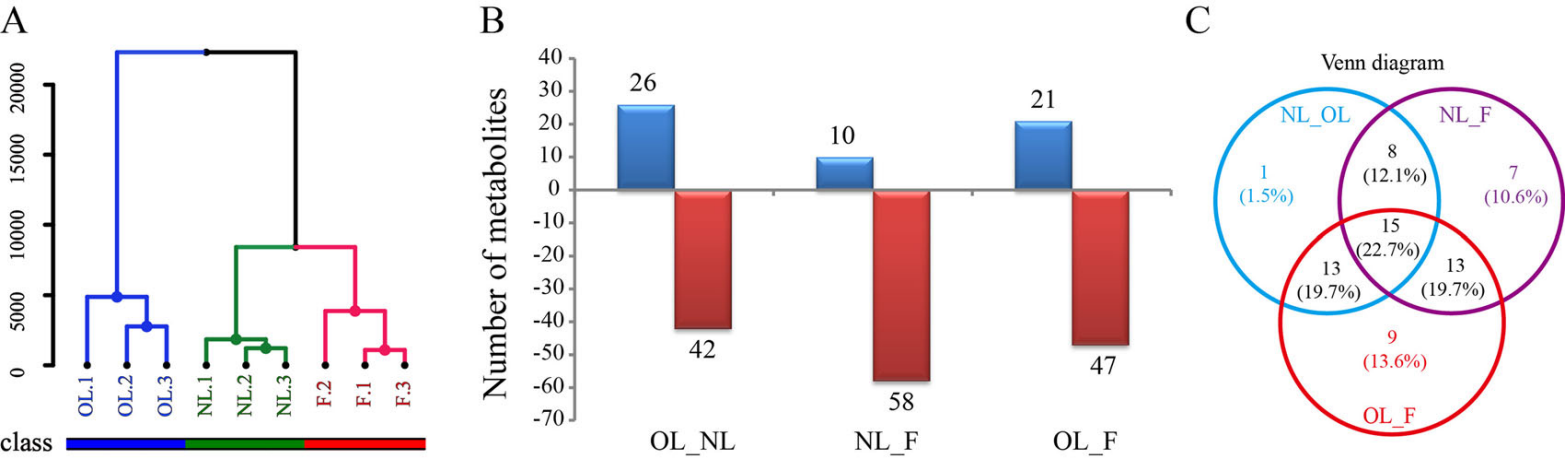
**Hierarchical cluster analysis.** (A) HCA of metabolites and contents comparison among samples NL, OL, and F in olives. (B) Comparison of metabolites among samples NL, OL, and F in olives, blue and red showed the high and low content between samples. (C) Venn diagram of metabolites among three comparison groups of olive leaves and fruit.

A hierarchical cluster analysis (HCA) (Kim et al., 2014) dendrogram was obtained using the metabolites detected in the leaves and fruits of olives (Fig. 2A). It is clear that samples from fully expanded leaves when olive began fruiting (NL), old leaves when olive fruits were maturated (OL), and maturated fruits (F) formed separate clusters. Samples of NL and OL were leaves collected at different times, while the HCA dendrogram did not show a similar relationship between the two leaf samples. Conversely, the samples of NL and F were clustered, suggesting metabolites in these samples were more similar. To analyze the contents of metabolites detected in different tissues, we compared all of the metabolites in each sample. The results revealed three groups, OL_NL (metabolite comparison between the samples of NL and OL), NL_F (metabolite comparison between the samples of NL and F), and OL_F (metabolite comparison between the samples of OL and F) (Table S1) had significant variation. In group OL_NL, 26 metabolites had higher contents in OL than NL, while 42 metabolites had lower contents in OL than NL. The 26 higher metabolites included nine organic acids, six polyols, three phosphates, and six sugars, while the 42 lower metabolites included 18 organic acids, four polyols, two phosphates, 11 sugars, and three fatty acids. In group NL_F, 10 metabolites had higher contents in NL compared to F, while 58 metabolites had lower contents in NL than F. The 10 higher metabolites included two organic acids, two phosphates, and four sugars. The 58 lower metabolites included 24 organic acids, 10 polyols, three phosphates, 13 sugars, and three fatty acids. In group OL_F, 21 metabolites had higher contents in OL than F, and 47 metabolites had lower contents in OL than F. The 21 higher metabolites included eight organic acids, two phosphates, and seven sugars. The 47 lower metabolites included 19 organic acids, 10 polyols, three phosphates, nine sugars, and three fatty acids (Fig. 2B).

We further analyzed the metabolite variations of each comparison group (NL_F, OL_F, and NL_OL), and a Venn diagram of these three groups showed that 15 (22.7%) metabolites were present in these groups (Fig. 2C). These metabolites included five sugars (trehalose, cellobiose, melibiose, 1-benzylglucopyranoside, erythrose), two polyols (digalactosylglycerol, galactosylglycerol), four organic acids (2,4,5-trihydroxypentanoic acid, glucaric acid, ribonic acid, 4-hydroxybenzoic acid), one phosphoric acid (glucose-6-phosphate), and one amino acid (pyroglutamic acid). Sugar and organic acid possessed most of the metabolites, which had different levels among the three samples, indicating dynamic changes in energy-related metabolites among new leaves, old leaves, and fruit. Thirteen (19.7%) metabolites were detected in both NL_F and OL_F, one sugar (arabinose), three polyols (threitol, myo-inositol, 2-methyl-1,3-butanediol), five organic acids (malonic acid, erythronic acid, alpha-ketoglutaric acid, 2,3-dihydroxybutanedioic acid, succinic acid), and three phosphoric acids (monomethylphosphate, nicotinic acid, glyceric acid). Additionally, 13 (19.7%) metabolites were detected in both NL_OL and OL_F: three sugars (2-O-glycerol-beta-D-galactopyranoside, maltose, lactose), one polyol (maltitol), and eight organic acids [oxalic acid, citric acid, trans-ferulic acid, 9-(Z)-octadecenoic acid, benzoic acid, pyruvic acid, 2-keto-L-gluconic acid, heptanoic acid]. Eight (12.1%) metabolites were detected in both NL_OL and NL_F: three sugars (sucrose, xylose, fructose), one polyol (glycerol), and four organic acids (malic acid, 2-methyl-fumaric acid, itaconic acid, 4-hydroxybutanoic acid). One (1.5%) metabolite was detected only in NL_OL, seven (10.6%) metabolites were detected only in NL_F, and nine (13.6%) metabolites were detected only in OL_F (Table 1).

**Table 1.**
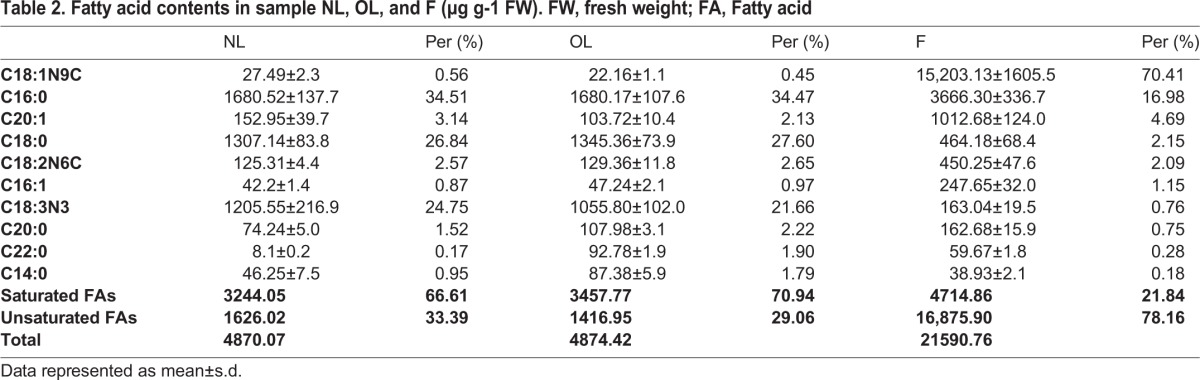
**Metabolites comparison in different samples**

### Metabolites correlation analysis among leaves and fruit

**Fig. 3.**
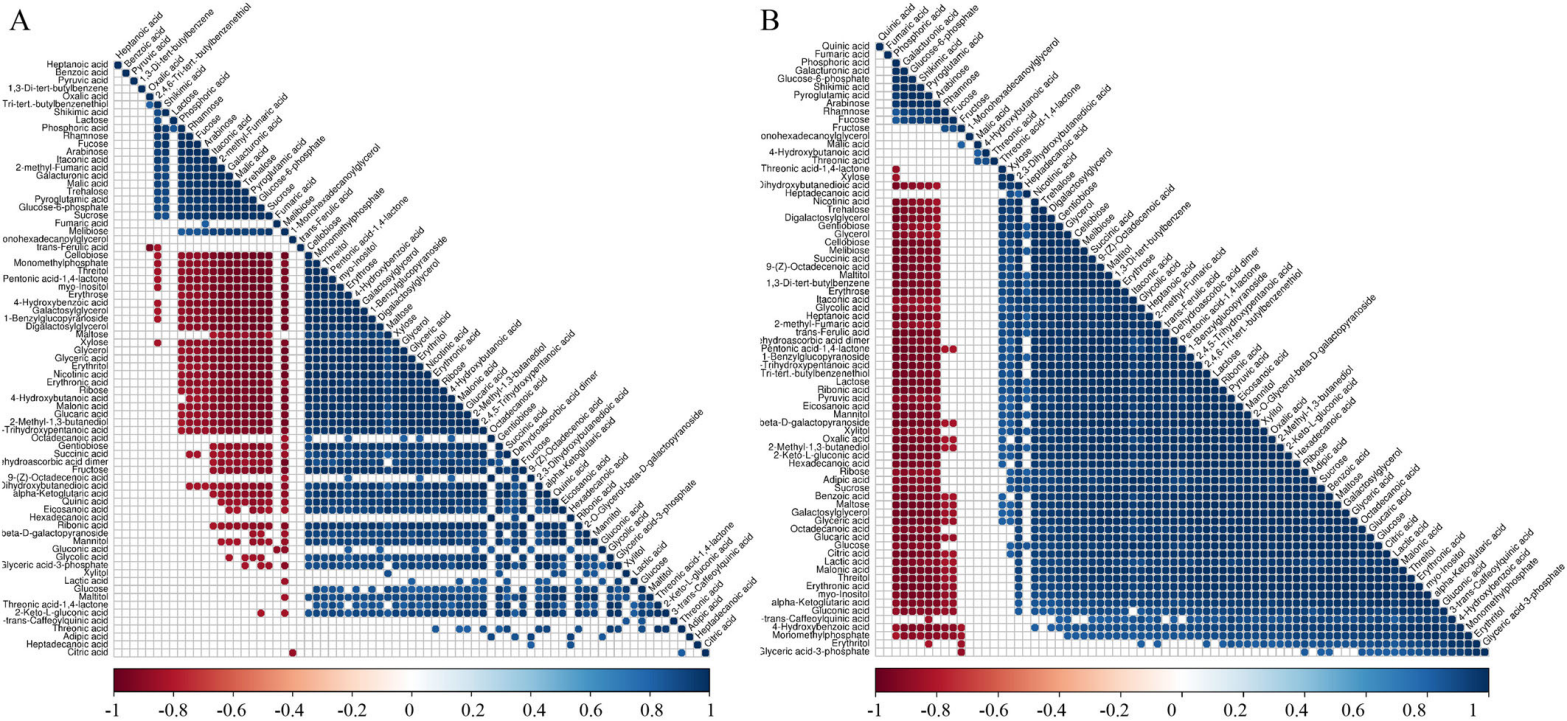
**Metabolite-metabolite correlation analysis.** Positive correlations are shown in blue; negative correlations are shown in red. (A) Metabolite-metabolite correlation of group NL_F. (B) Metabolite-metabolite correlation of group OL_F.

Metabolite-metabolite correlation analysis among the identified metabolites was conducted by Pearson correlation coefficient analysis in olive leaves and fruit (Rao et al., 2016). This analysis allowed the identification of metabolites that related to each other in tissues. Specifically, we compared metabolite correlations between each pair of samples (NL and F, OL and F, and NL and OL), and the metabolite-metabolite correlations of these three sample combinations showed unique profiles. In the NL and F group, a total of 5776 correlations were analyzed, among which 3136 resulted in significant correlation coefficients (*P*<0.1). Out of these 3136 significant correlations, 1918 were positive and 1218 were negative (Fig. 3A). Many organic acids and sugars, such as fructose, glucose, pyruvic acid, oxalic acid, heptanoic acid, malonic acid, and benzoic acid, were found to have negative correlations compared to other metabolites, and all of the fatty acids and most of the polyols had positive correlations compared to other metabolites. In the OL and F group, a total of 3025 metabolites had significant correlations, among which 2425 had positive and 600 had negative correlations. Organic acids, such as quinic acid, fumaric acid, galacturonic acid, and shikimic acid, had negative correlations compared to other metabolites (Fig. 3B). In the NL and OL group, a total 5184 metabolites had significant correlations, among which 3952 were positive and 1232 were negative (Fig. S1).

### Fatty acid metabolism in olive

Fatty acids, which are the main component of olive fruit, are usually unbranched compounds with an even number of carbons ranging from 12 to 22 and 0 to 3 cis double bonds. Moreover, unsaturated fatty acids accounted for over 95% of the total fatty acids. We conducted a targeted global metabolomics platform with GC-MS to verify the accurate content of fatty acids, and the results showed the same variation trends in fatty acids content detected by an untargeted method. A total of 30 FAs were identified, among which the contents of unsaturated FAs accounted for over 78% of the total in sample F (Table 2). Two dominant components, oleic acid (C18:1Δ^9c^, 70.41% of total FAs) and palmitic acid (C16:0, 16.98%), together accounted for over 86% of the total FAs in sample F. Four components also had moderate levels of total FAs, including cis-11-eicosenoic acid (C20:1Δ^11c^, 4.69%), stearic acid (C18:0, 2.15%), linoleic acid (C18:2Δ^9c, 12c^, 2.09%), and palmitoleic acid (C16:1Δ^9c^, 1.15%). Other minor FAs were also detected at trace levels, including α-linolenic acid (C18:3Δ^9c, 12c, 15c^, 0.76%), eicosanoic acid (C20:0, 0.75%), behenic acid (C22:0, 0.28%), and myristic acid (C14:0, 0.18%).

**Table 2.**
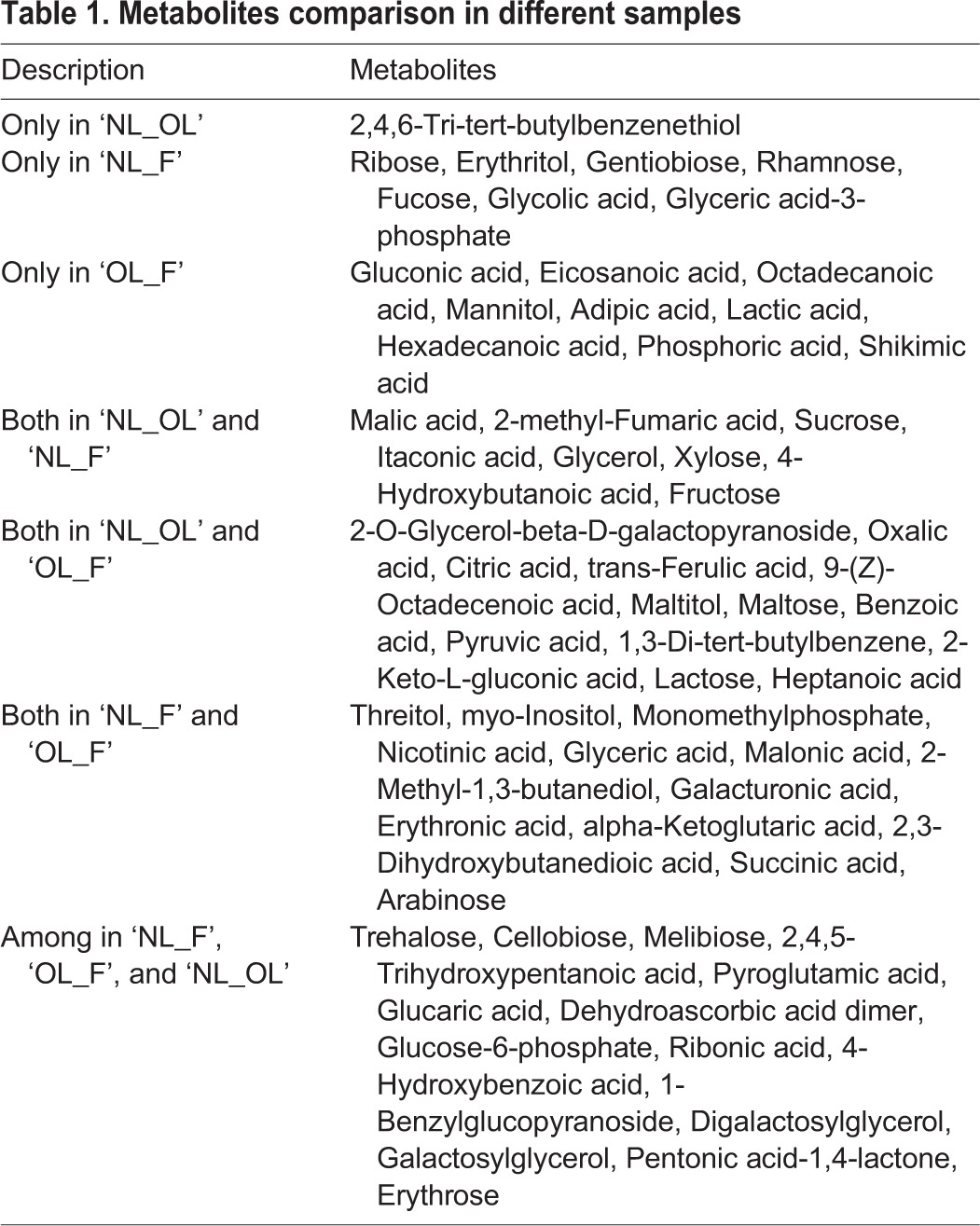
**Fatty acid contents in sample NL, OL, and F (µg g-1 FW). FW, fresh weight; FA, Fatty acid**

Unsaturated fatty acids content is an important parameter of different vegetable oils. Oleic acid is the predominant unsaturated fatty acid found in many plant oils required in the diet of higher animals, including humans. Analysis of the fatty acids composition of six Tunisian olive varieties showed a 32.37%–70.35% oleic acid content (Haddada et al., 2007). Analysis of the fatty acid profiles of 563 oil samples from 17 varieties in La Rioja (Spain) revealed three levels of oleic acid content, low (<55%), intermediate (55–65%) and high (>65%) (Rondanini et al., 2011). Olive oil forming four cultivars grown in two different geological areas was detected for the fatty acids contents, which were found to contain 66.05%–76.30% oleic acid (Rondanini et al., 2011). Fatty acids are also involved in plant tolerance to biotic and abiotic stresses through their effects on the fluidity of cell membrane (Upchurch, 2008). Linoleic acid is the most abundant fatty acid in plant membranes, and the moderate content of linoleic acid in the leaves and fruits suggests it may be involved in modification of the membranes fluidity during development of walnut kernels. Free α-linolenic acid has been shown to exert antifungal activity in many plants (Göbel et al., 2002; Walters et al., 2004; Sánchez-Sampedro et al., 2007). This compound is also a precursor of jasmonic acid, which plays a vital role in plant responses to biotic and abiotic stress (Wasternack, 2007; Wasternack and Hause, 2013). The level of α-linolenic acid in leaves (both in NL and OL) is much higher than in fruits, indicating that leaves are the main organ that provides an indicator of the fungal defense response during olive development. The saturated fatty acid palmitic acid was found to have the highest level in olive leaves and the second highest level in olive fruit. Analysis of the fatty acid composition of 224 samples of Cornicabra virgin olive oil collected in Spain during a series of crop seasons from 1995/1996 to 1999/2000 revealed that they contained 6.99%–11.05% palmitic acid (Aranda et al., 2004). Analysis of 475 Sicilian virgin olive oils produced in 10 different crop years (from 1993 to 2004) from four cultivars grown in two different geological areas revealed that they contained 10.00%–15.80% palmitic acid (Di Bella et al., 2007). Additionally, 16 virgin olive oils from 14 cultivars were found to contain 9.50%–23.09% palmitic acid (Gila et al., 2015). In the present study, fruit samples were found to contain 16.98% palmitic acid, which accounted for 77.75% of the saturated fatty acids in fruit, indicating that palmitic acid was the main saturated fatty acid in olive oil. Moreover, 34.51% palmitic acid was observed in new leaves, while 34.47% was observed in old leaves, suggesting that palmitic acid plays an important role in the metabolism of leaves.

### Metabolic pathways in different tissues of olive

**Fig. 4.**
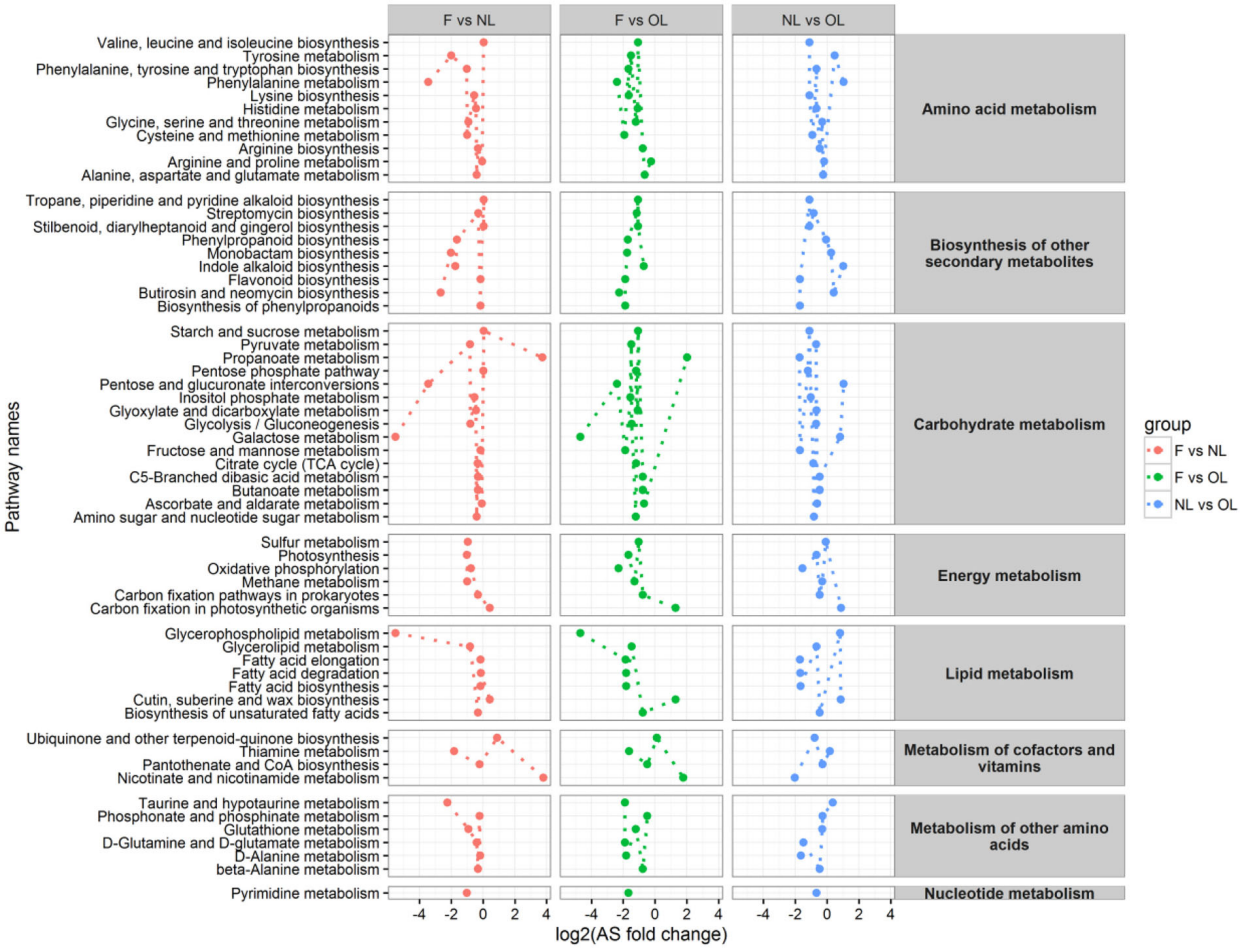
**Activities of olive metabolic pathways according to comparisons between samples.** Comparisons between NL_F, OL_F, and NL_OL are shown in red, green, and blue, respectively.

In this study, four major kinds of metabolites (sugar, fatty acid, polyol, and organic acid) were used to assess metabolic shifts among tissues. Most of the metabolic pathways were downregulated between the sample of fruit and new leaves, indicating that the majority of the metabolic pathway activity was relatively low. The metabolism of cofactors and vitamins was upregulated in this group, suggesting that metabolites related to this metabolism pathway were active. Similar results were obtained in the group of fruit and old leaves, in which most of the metabolic pathways were downregulated. Lipid metabolism and energy metabolism were upregulated, indicating fatty acids, which were the major lipid related metabolites, were activated between the fruit and old leaves. In the group of new leaves and old leaves, metabolic pathways were mostly up regulated, suggesting large metabolite shifts between these two tissues (Fig. 4).

Most subgroups of amino acids were downregulated between the sample of fruit and new leaves and the sample of fruit and old leaves. For example, tyrosine metabolism and phenylalanine metabolism were both upregulated in new and old leaves, indicating that their metabolism was activated in new leaves. However, among secondary metabolites, phenylpropanoid biosynthesis, monobactam biosynthesis, indole alkaloid biosynthesis, flavonoid biosynthesis, and butirosin and neomycin biosynthesis were downregulated between the fruit and new leaves, indicating that these metabolite biosynthesis pathways were also upregulated between the new and old leaves. Phenylpropanoids are indicators of plant stress responses to both variations in light and plant resistance to pests, and they contribute to all aspects of plant responses towards biotic and abiotic stimuli. Indole alkaloids have been shown to play roles in the defense against abiotic or biotic stresses and herbivory, or to be involved in chemical attractions to facilitate predation, pollination, or seed dispersal. Flavonoids play an important role in the interactions between plants and their environment, are involved in protecting plants from the harmful effects of UV irradiation, and play a crucial role in the sexual reproduction process (Koes et al., 1994). These secondary metabolites were upregulated in new leaves, indicating that metabolites involved in the resistance to biotic and abiotic stresses are mainly biosynthesized in new leaves. In the carbohydrate metabolism group, pentose and glucuronate interconversions and galactose metabolism were upregulated between the new and old leaves, suggesting that carbohydrates biosynthesized in new leaves provide energy resources for fatty acid metabolism during fruit development. In the lipid metabolism group, the glycerophospholipid metabolism was upregulated between the fruit and leaves (both new and old leaves), and fatty acid elongation, fatty acid degradation, and fatty acid biosynthesis were upregulated in new leaves relative to old leaves, suggesting that the high oil content of fruit was mainly biosynthesized and shifted from new leaves.

There are three general purpose technologies that have emerged as the primary workhorses in metabolomics: NMR spectroscopy; GC­MS; and liquid chromatography MS (LC­MS). NMR is well-suited to metabolomics studies as it can uniquely identify and simultaneously quantify a wide range of organic compounds in the micromolar range. NMR is nondestructive, so samples can continue for further analysis. However, the major limitation of NMR for comprehensive metabolite profiling is its relatively low sensitivity, making it inappropriate for the analysis of a large number of low-abundance metabolites. Currently The GC-MS method has been one of the most popular metabolomics techniques. GC-MS has a drawback in that only volatile compounds or compounds that can be made volatile after derivatization can be analyzed, and derivatization often requires extensive sample treatment. However, once the analysis is focused on low molecular weight metabolites, GC-MS is highly efficient, sensitive, and reproducible. Although chemical derivatization provides significant improvement in the GC separation of many compounds, it also can introduce artifacts due to the derivatization process itself. A significant advantage of GC–MS with electron ionization (EI) is the availability of many searchable mass spectral libraries.

## MATERIALS AND METHODS

### Plant materials, metabolite extraction, derivatization for GC-MS

Fully expanded leaves when olive began fruiting (NL), old leaves when olive fruits were maturated (OL), and maturated fruits (F) of four to five years old were used as representative olive cultivars and were sampled at the research garden of Research Institute of Forestry, Chinese Academy of Forestry in Gansu of China. All samples were frozen in liquid nitrogen immediately and stored at −80°C until further processing. Samples were ground under liquid nitrogen to obtain a fine powder, after which 100 mg of lyophilized powder per sample was weighed for metabolite extraction. Cold extraction was employed, and the extraction protocol was followed according to previous studies, with slight modifications (Weckwerth et al., 2004). Briefly, 100 mg plant samples and five steel balls were added into 5 ml centrifuge tubes which were then transferred into liquid nitrogen for 5 min. Samples were powdered by the high flux organization grinding apparatus; 1.4 ml of cold solvent (maintained at −20°C) comprised of methanol:chloroform:water in a ratio of 5:2:1 together with 50 µl of internal standard (Ribitol stock concentration, 0.2 mg/ml) was added to the ground material and vortexed for 10 s. The mixtures were then kept on ice for 25 min with intermittent vortexing or shaking for 10 s. The homogenate was subsequently centrifuged at 13,000×***g*** and 4°C for 10 min, after which the supernatant was transferred to a new tube with 200 µl chloroform and 500 µl of deionized water and vortexed again. The mixture was then centrifuged at 13,000×***g*** and 4°C for 5 min. Next, the solution was separated into the upper (aqueous) and lower (organic) phases. The aqueous phase was then dried under vacuum and derivatized for GC-MS analysis. The derivatization steps were as follows. The dried aqueous phase was dissolved in 20 µl of methoxyamine hydrochloride (M.HCL) solution (40 mg/ml in pyridine), then kept at room temperature for 2 h. Subsequently, 80 µl of N-Methyl-N-(trimethylsilyl) trifluoroacetamide (MSTFA) was added to the M.HCL mixture and incubated at 37°C for 30 min with shaking. The derivatized samples were then centrifuged at 13,000×***g*** for 5 min, after which they were transferred to autosampler vials for GC-MS analysis. Furthermore, a mixed n-alkane standard solutions C8–C20 and C21–C40 (Sigma Aldrich) was used for the determination of the retention indices (RI). The sample set also included a quality control (QC) sample consisting of an aliquot (40 µl) of a mixture of all prepared sample extracts.

### Untargeted metabolomic analysis

The derivatized samples were analyzed using a global unbiased mass spectrometry-based platform with GC-MS, and data normalization was performed according to a previous study (Lawton et al., 2008). The samples were randomized and the data acquisition was conducted in one batch to eliminate system errors. GC-MS was conducted using an Agilent 7890A/5975C GC-MS and an auto-sampler unit. An HP-5MS (Agilent J&W Scientific, Folsom, CA, USA) column with a thickness of 0.25 µm, diameter of 250 µm, and length of 30 m was used to separate derivatized metabolites. A 1-µl aliquot of sample was injected in split mode in a 1:20 split ratio by the auto-sampler. The injection temperature was set at 280°C and the column oven temperature was 80°C, with helium as the carrier gas. The mass spectrometry settings were as follows: ion source temperature, 250°C; interface temperature, 280°C; solvent cut time, 5 min. For analysis, the temperature program was: 5 min hold at 40°C, followed by 10°C/min ramp to a final temperature of 300°C, which was held for 5 min. The scan range was 35–750 m/z. PCA was performed using the R software (www.r-project.org). Heatmap packages available in R were used to draw heat maps, and the Mev (MultiExperiment Viewer) 4.8 software was used to perform one-way ANOVA with standard Bonferroni correction. Identified metabolites were mapped onto general biochemical pathways according to the annotation in KEGG (Kyoto Encyclopedia of Genes and Genomes). Metabolic network maps were constructed by incorporating the identified and annotated metabolites using Cytoscape 3.2.0 (www.cytoscape.org/). Activities of olive metabolic pathways were determined based on metabolomic data from all three samples. The activity scores (AS) for each pathway were calculated using our Pathway Activity Profiling (PAPi) algorithm (Han et al., 2012).
